# Concrescence: Cone-Beam Computed Tomography Imaging Perspective

**DOI:** 10.1155/2016/8597872

**Published:** 2016-10-09

**Authors:** Ali Zakir Syed, LeelaSubhashini Choudary Alluri, Dhiraj Mallela, Troy Frazee

**Affiliations:** ^1^Department of Oral and Maxillofacial Medicine and Diagnostic Sciences, CWRU School of Dental Medicine, Cleveland, OH, USA; ^2^Oral and Maxillofacial Surgery, Western Reserve OMS, Middleburg Heights, OH, USA

## Abstract

Concrescence is a form of twinning, formed by the confluence of cementum of two teeth at the root level. The diagnosis of concrescence has largely relied on the conventional 2D imaging. The 2D imaging has inherent limitations such as distortion and superimposition. Cone-Beam CT eliminates these limitations. The aim of this article was to describe a case of dental abnormality using Cone-Beam CT imaging modality. Volumetric data demonstrated confluence of left mandibular third molar with a paramolar, a supernumerary tooth. To our knowledge, this is the second case in the dental literature reported demonstrating the use of Cone-Beam CT in the diagnosis of concrescence.

## 1. Introduction

Concrescence is a developmental anomaly of the teeth, wherein roots fuse, with no evidence of periodontal space between two or more normal teeth below the cementoenamel junction. It is caused by a confluence of the cemental surfaces [1–3]. The confluence may occur in between normal tooth and supernumerary tooth [2]. The prevalence of concrescence is reported to be highest in the posterior maxilla [4]. Concrescence classically affects maxillary molars, mostly maxillary second and third molars [4]. According to the latest studies, extracted teeth show frequency of concrescence to be 0.8% in adult teeth and in deciduous teeth to be 0.2–3.7% [5]. Some reported complications of concrescence include periodontal destruction [6]. Extractions of these teeth may be difficult due to large mesiodistal dimensions and could result in the alveolar bone fracture and tooth fracture or can cause sinus opening [6]. Moreover, placement of a rubber dam clamp for isolation during the endodontic procedure could be challenging as well [6]. Various conventional two-dimensional imaging techniques like periapical, bitewing, occlusal, and panoramic radiographs are commonly used in routine dental practices. However, these two-dimensional imaging techniques may pose challenges to diagnostic task oftentimes because of overlap and superimposition [4]. Cone-Beam Computed Tomography (Cone-Beam CT) was developed in the 1990s and it is an addition to the imaging armamentarium for use in diagnosis. It has relatively low dose compared to MDCT [7–9]. To our knowledge, this is the second case report describing the concrescence dental anomaly in which CBCT imaging was used for evaluation.

## 2. Case Report

**Figure 1:**
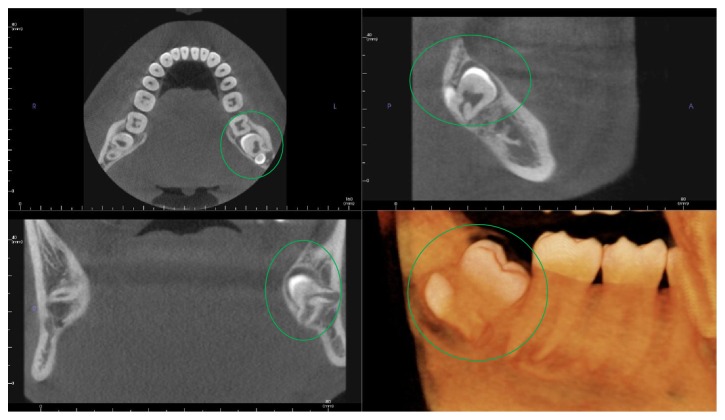
MPR view showing concrescence.

**Figure 2:**
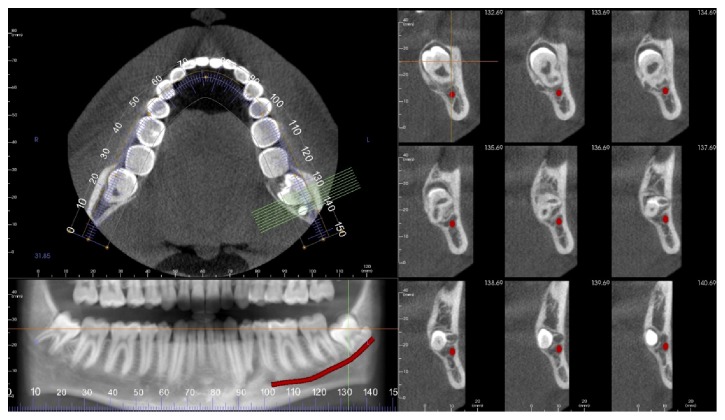
Panoramic radiograph and cross-sectional view proximity of the inferior canal to concrescence tooth.

**Figure 3:**
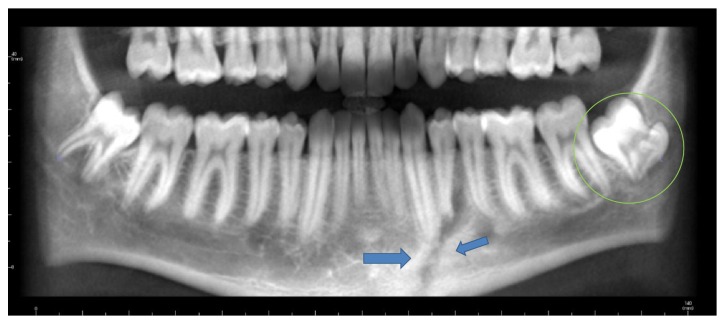
Panoramic radiograph showing concrescence in the lower left third molar region (green circle); additionally, fracture line (blue arrows) is noted in the premolar region (left side).

**Figure 4:**
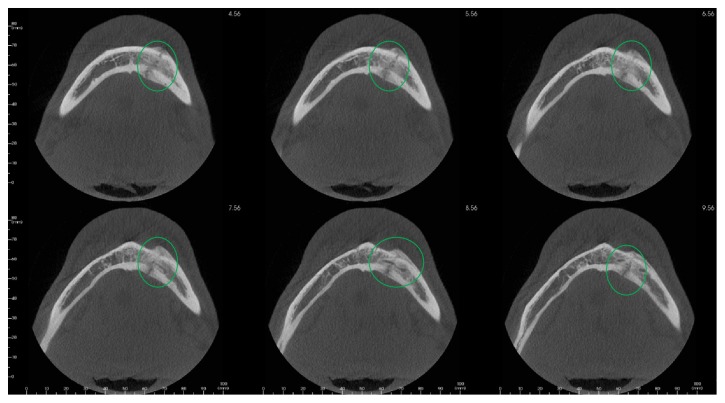
Axial view demonstrating osteomyelitic reaction.

An 18-year-old male's CBCT scan was performed for the evaluation of erupting the third molar. History of trauma was reported 4 months back in the left mandibular region. The CBCT volume was obtained with the CS9300 unit (Carestream, Atlanta, GA, USA) at a private dental office. The parameters used to acquire this scan are 90 Kv, 4 mA, and 9 × 9 field of view scan. Medium FOV was obtained to evaluate both trauma and dental related abnormality. The DICOM data was sent for evaluation via compact disc. The data was evaluated by board-certified oral and maxillofacial radiologist (SAZ) and volumetric data showed erupting # 17. The distolingual aspect of # 17 exhibited possible supernumerary tooth that appeared to be fused with # 17 at the root level (Figure 1). The radiographic impression of concrescence was made. This anomaly could complicate the extraction procedure because of fusion with the root and the proximity of the tooth to the inferior alveolar canal (Figure 2). Additionally, an old fracture line was noted extending from the mid alveolar region in the premolar region (Figure 3). The old fracture shows nonunion and osteomyelitis (Figure 4). The patient was referred for appropriate treatment.

## 3. Discussion

Tooth related abnormalities include morphological changes such as a change in size, shape, and a number of the teeth [10]. Most of the anomalies are genetic with multifactorial etiology [11]. The most important anomaly associated with a number is supernumerary teeth. Supernumerary teeth may occur as either single tooth or mutiple teeth; they may be either unilateral or bilateral and in the maxilla or in the mandible. The most important anomalies of shape are gemination, fusion, and concrescence which could simulate dental twinning anomalies. Concrescence may take place with another tooth or with supernumerary tooth [1, 2]. Concrescence is of two types, true concrescence that occurs during root formation and acquired type concrescence that occurs after root formation is complete [12]. There is no predilection towards any race, age, gender, and primary or permanent teeth [4]. Although it is very difficult to find out the exact etiology for concrescence, local trauma, space restriction during development, excessive occlusal force, or local infection after development may be the suspected causative factors [4]. Concrescence usually involves posterior maxilla [4]. However, in our case report, it involved mandibular left posterior region. In our case as per patient history, concrescence may be acquired due to trauma. Additionally, clinically detection of concrescence is nearly impossible. There are numerous conventional two-dimensional imaging techniques, having a major limitation such as superimposition or overlap [4]. CBCT would be a powerful adjunct in capturing three-dimensional images [9]. In our case, the practitioner must consider presence of concrescence proximity very closely to inferior alveolar canal and make the appropriate modifications to surgical technique in order to prevent any undesirable surgical complications.

## 4. Conclusion

This case report illustrates the importance of CBCT 3D imaging technique in evaluating and accurately diagnosing dental abnormalities and the proximity to the inferior alveolar canal.
